# *SERPINA1* is a direct estrogen receptor target gene and a predictor of survival in breast cancer patients

**DOI:** 10.18632/oncotarget.4441

**Published:** 2015-06-29

**Authors:** Hei Jason Chan, Haiqing Li, Zheng Liu, Yate-Ching Yuan, Joanne Mortimer, Shiuan Chen

**Affiliations:** ^1^ Department of Cancer Biology, Beckman Research Institute of the City of Hope, Duarte, California, USA; ^2^ Department of Molecular Medicine, Beckman Research Institute of the City of Hope, Duarte, California, USA; ^3^ Bioinformatics Core Facility, Beckman Research Institute of the City of Hope, Duarte, California, USA; ^4^ Department of Medical Oncology, Beckman Research Institute of the City of Hope, Duarte, California, USA

**Keywords:** estrogen receptor, breast cancer, endocrine resistance, SERPINA1, survival analysis

## Abstract

Of all breast cancer patients, about 70% are ER+ and 10% are ER+/HER2+. The ER+/HER2+ patients have a worse outcome compared to ER+/HER2- patients. Currently there is a lack of effective prognosis biomarkers for the prediction of outcome in ER+/HER2+ patients. Genome-wide differences in ER binding between the endocrine-responsive and endocrine-resistant cells were discovered using ChIP-seq, and combined with gene expression microarray data to identify direct ER target genes. These genes were correlated to survival outcome using publicly available breast cancer patient cohorts. We found the expression of the gene *SERPINA1* to have a significant predictive value for the overall survival (OS) of ER+ patients in the TCGA cohort, and validated this finding in the Curtis cohort. *SERPINA1* also has a significant predictive value for the OS of ER+/HER2+ patients in the TCGA cohort, with validation in the Bild cohort. The expression of *SERPINA1* can be suppressed by fulvestrant and HER2 siRNA. Our results indicate that ER is constitutively activated, resulting in an E2-independent ER binding to the *SERPINA1* gene and upregulation of *SERPINA1* expression. Importantly, results of survival correlation suggests that high expression of *SERPINA1* could be predictive for a better clinical outcome of ER+ and ER+/HER2+ patients.

## INTRODUCTION

The estrogen receptor α (ER) is a crucial transcription factor that is required for cell proliferation in the majority of breast cancer cases, which accounts for about 70% of all breast cancers. A major treatment of ER+ breast cancer is endocrine therapy using anti-estrogens like tamoxifen or aromatase inhibitors (AIs). However, a significant number of ER+ patients are not responsive to such treatment (i.e., *de novo* resistance) and some patients develop resistance during endocrine therapy (i.e., acquired resistance). Previous studies in our lab have shown that the ER is required for growth in both endocrine (therapy)-responsive and endocrine-resistant breast cancer cells, but only endocrine-responsive cells require estrogen for the proliferation [1]. The global genomic binding profile of ER has been well documented in endocrine-responsive breast cancer cells but not in endocrine-resistant cells [2]. To investigate the molecular action of AIs, our laboratory has generated an aromatase-overexpressing MCF-7 cell line, i.e., MCF-7aro [3]. For this project, we used MCF-7aro cells as a model for endocrine-responsive breast cancer, and Long Term Estrogen Deprived (LTEDaro) cells as a model for endocrine-resistant breast cancer [4]. In the endocrine-responsive breast cancer cells, 17β-estradiol (E2) acts as a ligand and binds to ER, activating the ER and causing its translocation from the cytosol to the nucleus. The E2-bound ER then binds to the chromatin to regulate the expression of target genes. In the endocrine-resistant cells, the ER can be activated by other mechanisms such as phosphorylation, so even in the absence of E2, the ER is able to bind to chromatin and activate target genes. The ligand-independent activation of ER is thought to play key roles in endocrine-resistant breast cancer because the ER degrader, fulvestrant (ICI 182, 780), is able to suppress the expression of ER-regulated genes [5]. The goal of this study is to find such ER binding sites and target genes which will improve our understanding of the roles of ER in both endocrine-responsive and resistant cancers.

To better understand the physiological action of ER-target genes, we used chromatin immunoprecipitation with next-generation sequencing (ChIP-seq) as a tool to identify differences in ER binding between endocrine-responsive and endocrine-resistant cell lines in a genome-wide manner. In previous studies, our lab has performed Affymetrix GeneChip genome-wide microarray gene expression analysis to detect differentially estrogen-regulated genes [1], but these target genes include direct and also indirect ER targets. The combination of ChIP-seq with microarray gene expression analyses allows us to identify the direct ER target genes. We had hypothesized that the identification of such genes could allow us to find a gene that acts as a biomarker associated with endocrine response of breast cancer, which would be valuable for the prediction of the consequence of endocrine therapy and could facilitate the selection of the most effective treatment options for patients. Our extensive ER ChIP-seq analysis has resulted in a candidate gene *SERPINA1* that has a clear ER binding site in its promoter region and higher expression level in LTEDaro DMSO (i.e., in the absence of E2). The levels of *SERPINA1* mRNA have been found to be significantly higher in LTEDaro DMSO than in E2-treated MCF-7aro E2 [1]. Based on our survival analysis results using the publicly available large panel of The Cancer Genome Atlas (TCGA) 779 breast cancer patient cohort [6] with clincopathological information, we further hypothesize that the upregulation of *SERPINA1* in endocrine-resistant cells requires HER2 and has significant association with better survival outcome for ER+/HER2+ breast cancer.

*SERPINA1*, also known as α1-AntiTrypsin (AAT), is a protease inhibitor that can act on a variety of targets such as serine proteases. It has been demonstrated that *SERPINA1* expression can be stimulated by E2 in MCF-7 cells, and high expression of this protein inhibits colony formation [7]. *SERPINA1* has been proposed as a biomarker for various diseases such as Cutaneous Squamous Cell Carcinoma [8], Hepatitis B [9], insulinomas [10], NSCLC [11], papillary thyroid carcinoma [12] lung cancer [13] and breast carcinoma [14–16]. Unexpectedly, our results allow us to hypothesize that the single gene *SERPINA1* is a significant predictor of survival in ER+ and ER+/HER2+ breast cancer patients. Patients with ER+/HER2+ breast cancer generally have a worse outcome compared to ER+/HER2- patients [17, 18]. Currently there is no known predictive marker for the treatment outcome of ER+/HER2+ breast cancers [19], thus the ability of *SERPINA1* to predict the survival of this intrinsic subtype of breast cancer patients is valuable.

## RESULTS

### Bioinformatics analysis of ER ChIP-seq and microarray expression data

**Figure 1:**
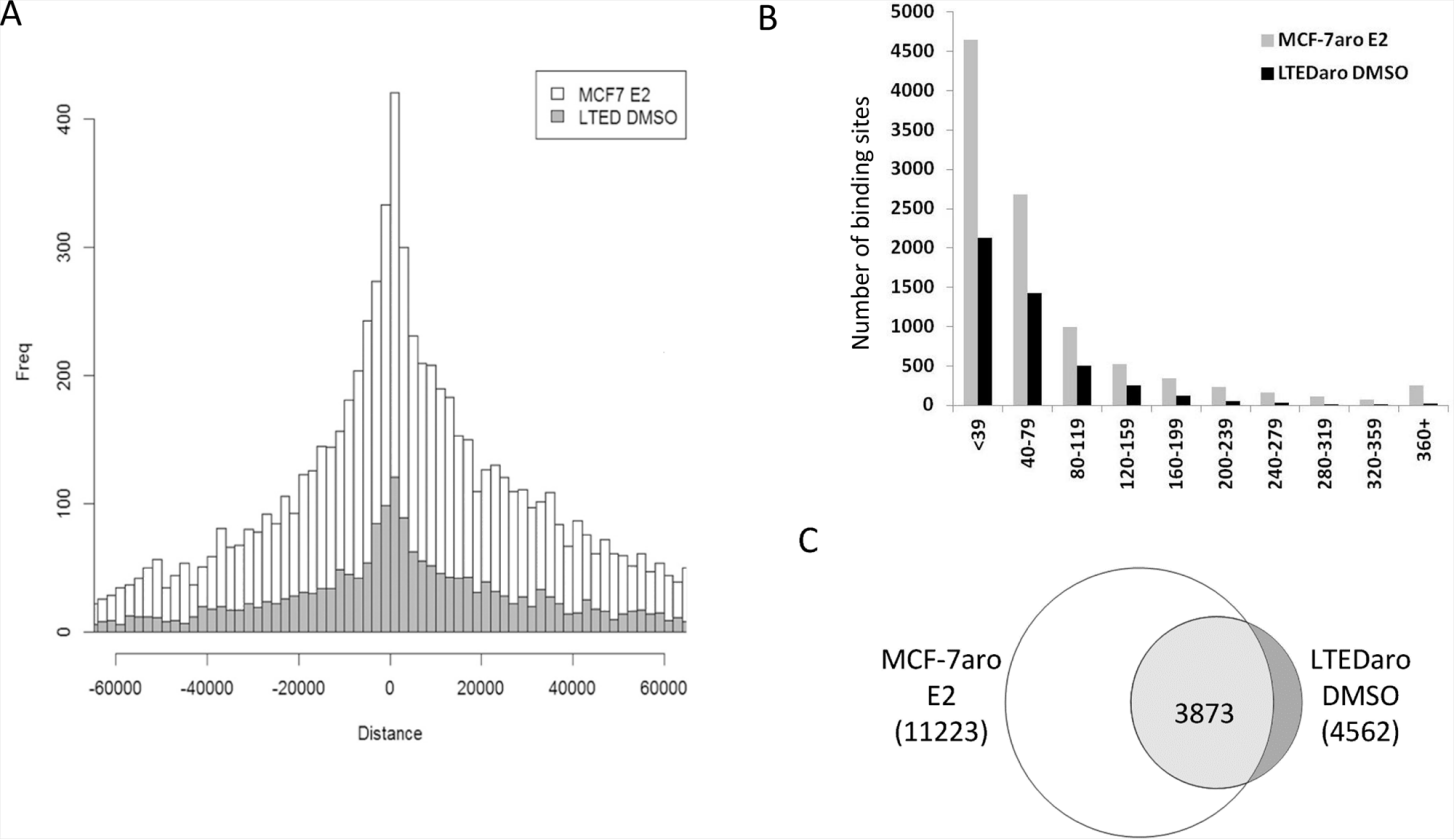
ER is able to bind chromatin independently of E2 in LTEDaro cells **A.** Distribution of ER binding sites relative to the closest TSS. MCF-7aro DMSO binding sites are almost evenly distributed and MCF-7aro E2 binding sites are more abundant close to the TSS. LTEDaro DMSO binding sites are abundant close to the TSS with a similar trend as the MCF-7aro E2. **B.** Correlation between the number of binding sites and binding intensities of LTEDaro DMSO and MCF-7aro E2. **C.** Comparison of LTEDaro DMSO and MCF-7aro E2 shows a majority of ER binding sites occur at the same location, although the intensities may be different.

The ER binding sites were annotated with the genes, and we plotted the number of binding sites against the distance to the closest transcription start sites (TSS). Comparison of the number of ER binding sites close to the TSS demonstrated that the distribution of the number of binding sites in the LTEDaro DMSO was comparable to that found in the MCF-7aro E2 (Figure 1A). This confirms that the ER binding in MCF-7aro is dependent on estrogens as expected, and most importantly, significant ER binding can occur without any hormones in LTEDaro cells. Analysis of the correlation between the number of binding sites and binding intensities also demonstrated that both LTEDaro DMSO and MCF-7aro E2 had a comparable normal distribution (Figure 1B).

Based on the overlap analysis of ER binding sites described in Materials and Methods section, we performed a comparison between ER binding sites in hormone-independent LTEDaro DMSO and hormone-dependent MCF-7aro E2 cells, as shown in Figure 1C. A majority of the binding sites were in the common group, but it should be emphasized that although the common sites shared the same location, the ER binding intensities were not always similar between LTEDaro DMSO and MCF-7aro E2.

**Figure 2:**
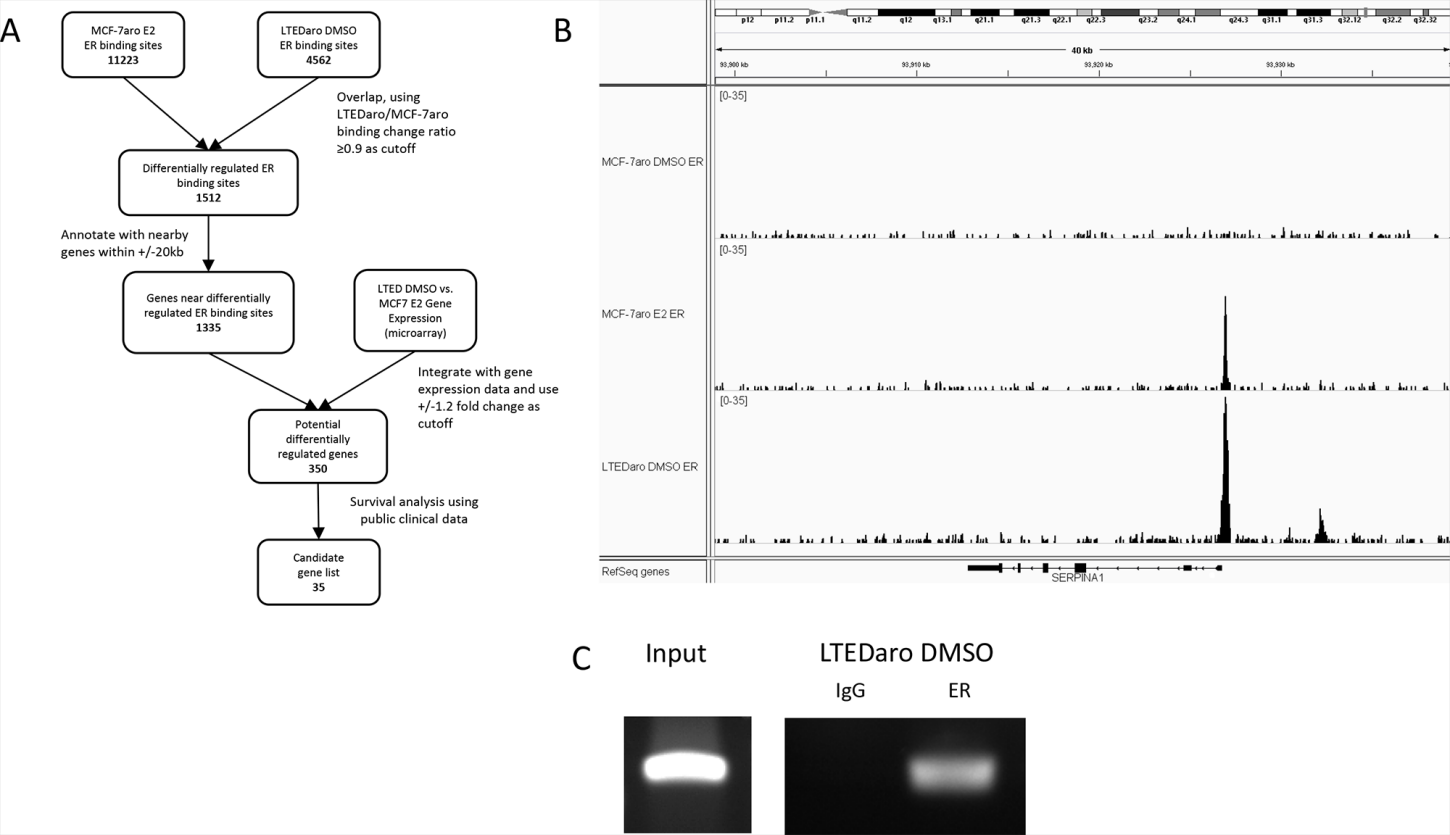
Identification of *SERPINA1* as an ER target gene with a distinctive ER binding site in the promoter region **A.** Summary of bioinformatics analysis of Illumina ChIP-seq and Affymetrix GeneChip gene expression microarray data, leading to the discovery of *SERPINA1* and its potential regulation by ER and HER2. **B.** *SERPINA1* has an ER binding site proximal to the TSS with higher level of ER binding in the LTEDaro DMSO cells compared to MCF-7aro E2 cells. In contrast, other well-known ER target genes have higher level of ER binding in the MCF-7aro E2 cells instead. **C.** ChIP PCR validation of ER binding site proximal to the TSS of *SERPINA1* confirms the ER binding in LTEDaro, as detected by ChIP-seq.

Figure 2A shows a comprehensive analysis workflow to determine the 350 differentially regulated genes annotated for further validation using large patient cohorts with survival information.

### Survival analysis using publicly available breast cancer patient cohorts

To determine the physiological significance of ER-binding genes in endocrine resistant cells, the resulting list of 350 genes was further analyzed for the ability to predict patient survival using the TCGA breast cancer patient cohort. 2-means clustering was adopted to cluster patients into high and low risk subgroups based on the 350 genes. As a whole, the group of 350 genes did not have a significant predictive value (data not shown). As described in the Materials and Methods section, using a Cox score cutoff of 2.39, the panel of 35 genes was further filtered for better survival correlation.

These 35 genes were then inspected individually in the IGV genome browser for ER binding site quality in order to narrow down the candidates for further qPCR validation and survival analysis. As described above, we are interested in ER binding sites that are dependent on hormones in the MCF-7aro cells but have significant ER binding in the LTEDaro DMSO cells. During the visual inspection we took into account the overall intensity of ER binding, the distance of the binding from the TSS, the ratios of MCF-7aro E2 to MCF-7aro DMSO binding, and ratios of LTEDaro DMSO to MCF-7aro E2 binding. Based on these criteria, we selected 3 genes with negative Cox score and 8 genes with positive Cox score from the panel of 35 genes. The 11 genes were then correlated with survival in ER+ and ER- patients using Kaplan Meier survival analysis. Based on *p*-values and biological relevance, we decided to focus on our analysis on the best candidate with strong ER binding in LTEDaro DMSO, the *SERPINA1* gene from the negative Cox score group. *SERPINA1* was brought to our attention because it has been reported to be an ER-regulated gene in breast cancer cells [29]. In our analysis, *SERPINA1* has a well-defined ER binding site with the distinctive property that ER binding in LTEDaro DMSO was found to be stronger than MCF-7aro E2 (Figure 2B). According to our previous Affymetrix microarray data, the expression level of this gene was about 3.4 fold higher in LTEDaro DMSO compared to MCF-7aro E2, and this difference is significantly higher according to our qPCR analysis (Figure 4A). Further literature search reveals that *SERPINA1* is a known marker for good prognosis in cancer [12, 14]. As indicated by a negative Cox score, a higher expression of *SERPINA1* was found to associate with better patient survival outcome in the TCGA large patient cohort, and validated using the Curtis and Bild breast cancer patient cohorts. These results will be discussed in more detail in the Kaplan Meier analyses. Before we performed the detailed survival analyses, we confirmed the *SERPINA1* ER binding and gene expression regulation by ER and HER2.

### The promoter of *SERPINA1* has an ER binding site

Although E2 was reported to up regulate the expression of *SERPINA1* twenty years ago [7], the mechanism was unknown at that time. A direct ER-mediated regulation of its expression in MCF-7 cells was reported by Simpson et al. [29], and it was reported that E2 addition did not significantly enhance the ER binding to the ERE in the promoter of *SERPINA1*. Our ER ChIP-seq analysis confirms that the *SERPINA1* gene has an ER binding site within the promoter region which overlaps with the TSS (Figure 2B), and the full ERE motif was found within this binding site by mapping known motifs (Supplementary Figure 1), which agrees with the previous study by Simpson et al [29]. In the MCF-7aro cells, the binding of ER to this site is dependent on estrogen, but in the LTEDaro DMSO cells, the ER binding has a higher intensity than MCF-7aro E2 even without estrogen. The ChIP PCR validation confirms the binding in LTEDaro DMSO (Figure 2C).

### *SERPINA1* promoter is E2 responsive

**Figure 3:**
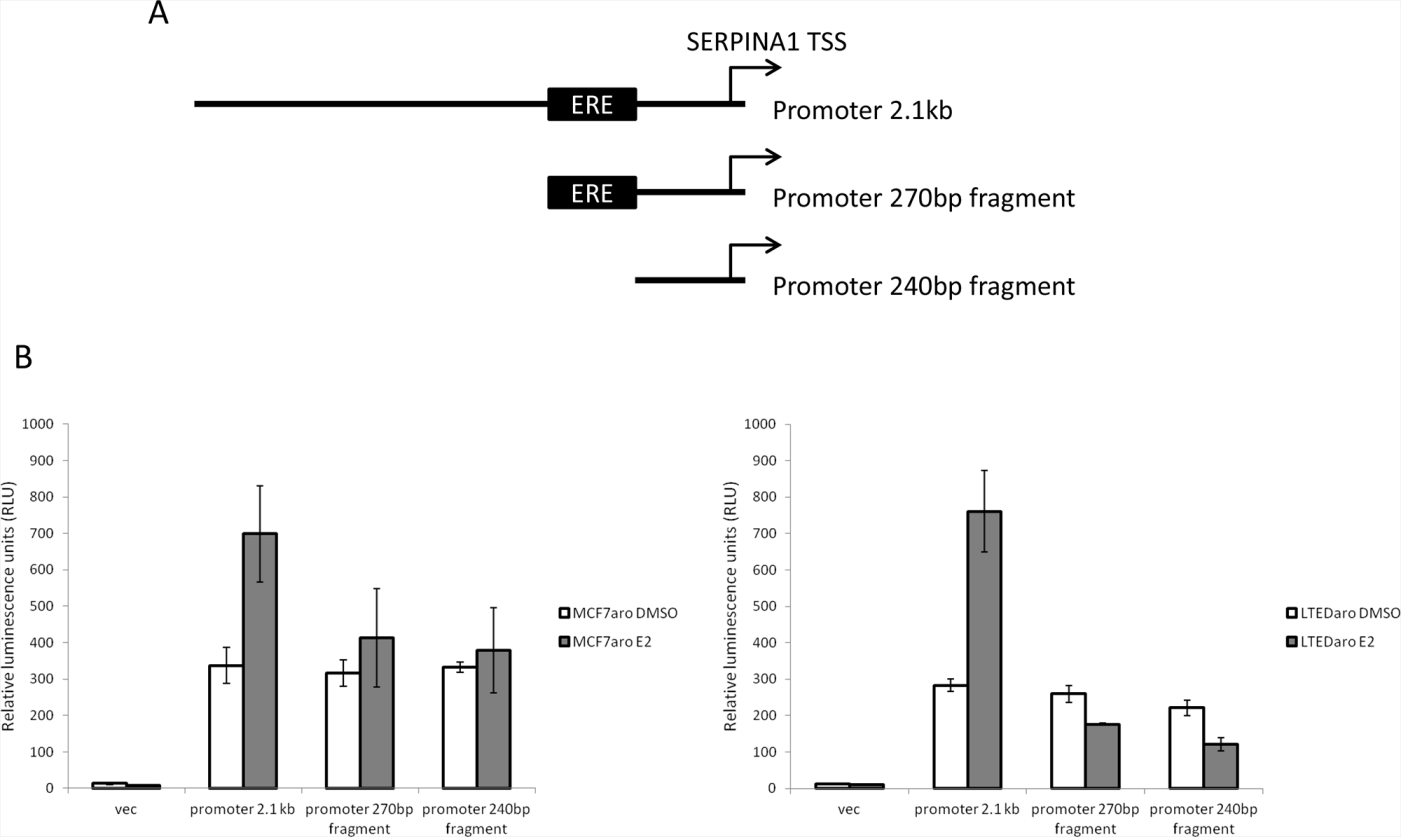
Cloning of *SERPINA1* promoter and deletion mutants into luciferase reporter vectors and validation of activity **A.** The *SERPINA1* full length promoter and two deletion mutants, with and without ERE, were cloned into luciferase reporter vectors. **B.** Luciferase activity assays of the reporter constructs in MCF-7aro and LTEDaro cells with E2 treatment shows E2 responsive transcriptional activation in the full length promoter.

To validate the activity and E2 response of the *SERPINA1* promoter, we cloned the full length and deletion mutants as described in the Materials and Methods section (Figure 3A). From the ChIP-seq peak calling data, the ER binding site covers a region approximately 2 kb which overlaps with the TSS. We cloned the region that covers the TSS and upstream 2 kb region, with a total length of 2.1 kb. This promoter construct treated with DMSO control showed a basal level of luciferase reporter activity compared to the empty vector control (Figure 3B). Upon E2 treatment, the luciferase activity increases, demonstrating that the full length promoter transcriptional activity is inducible by E2. In the truncated promoter fragments with and without the predicted ERE sequence, we observed a basal activity similar to the full length promoter treated with DMSO. With E2 treatment, we expected to see increased luciferase activity in the promoter fragment containing the ERE, but not in the ERE deleted fragment. Our results show that with E2 treatment, there is no significant increase in SERPINA1 expression in both constructs. This suggests that the proposed “ERE” alone is not sufficient, and there may be other sequences upstream of the predicted ERE that are needed for the E2 response.

### ER-dependent and HER2-dependent regulation of *SERPINA1* expression in endocrine-responsive and -resistant cells

**Figure 4:**
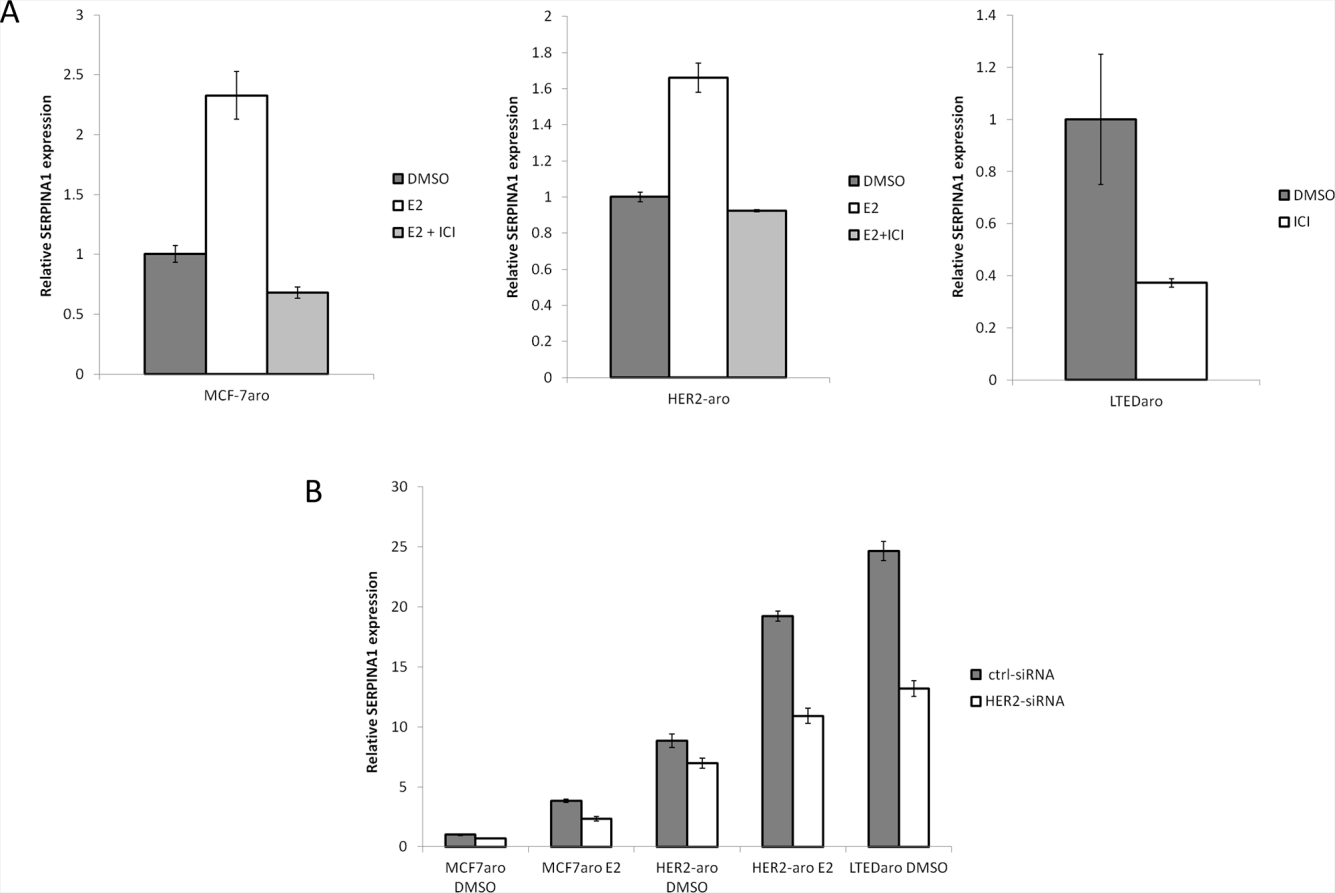
E2 and HER2 regulate the expression of *SERPINA1* through the ER **A.** Gene expression analysis of *SERPINA1* by qPCR shows that *SERPINA1* expression in MCF-7aro E2, HER2-aro E2, and LTEDaro DMSO can be suppressed by ICI treatment. **B.** Comparison of the *SERPINA1* expression in the control-siRNA treated cells shows that HER2-aro and LTEDaro cells have a higher expression compared to MCF-7aro. siRNA knockdown of HER2 shows that *SERPINA1* is downregulated by about 40% when HER2 levels are reduced.

In the MCF-7aro, LTEDaro and HER2-aro cell lines we examined, the *SERPINA1* expression is up regulated with E2 treatment and suppressed by the ER degrader, fulvestrant (ICI 182, 780) (Figure 4A). A search for *SERPINA1* in the Gene Expression Omnibus (GEO) database provided support that *SERPINA1* expression is stimulated by E2 in an ER-dependent manner [30, 31], and unexpectedly, by HER2 [32]. Since ER is known to be activated through ER-HER2 crosstalk in ER+/HER2+ breast cancer cells, we performed experiments to determine whether the expression of *SERPINA1* could be regulated by HER2. The expression level of *SERPINA1* in two tested HER2-overexpressing cell lines, HER2-aro [5] and LTEDaro cells [33], was found to be significantly higher than that in MCF-7aro cells, demonstrating that *SERPINA1* is a HER2 regulated gene (Figure 4A). The HER2-dependent regulation of *SERPINA1* expression was confirmed further by the down regulation of its expression by the treatment of siRNA targeting HER2 (Figure 4B). In attempt to further support our findings in additional cell lines, we checked the *SERPINA1* expression level in BT474 and MDA-MB-361cells, which express both ER and HER2 endogenously. The results show that E2 and ICI treatment did not significantly change *SERPINA1* levels, although there may be a weak induction by E2 in BT-474 cells (Supplementary Figure 2).

### Significance of *SERPINA1* expression in ER+ and ER+/HER2+ breast cancer

**Figure 5:**
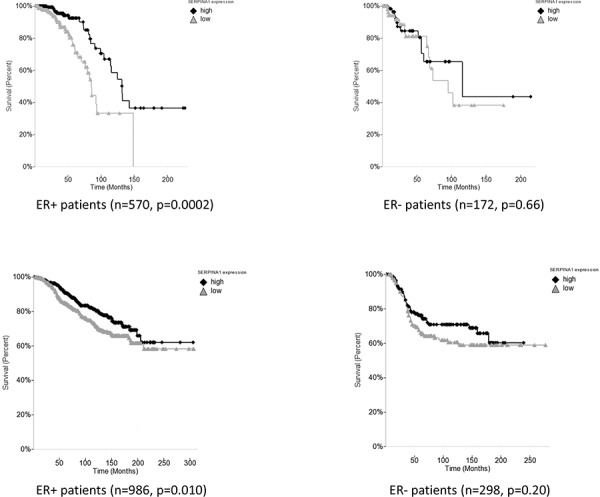
*SERPINA1* expression level is a predictive marker for ER+ breast cancer patient survival **A.** The survival analysis in TCGA breast cancer cohort of ER+ and ER- patients shows that *SERPINA1* has a significant predictive value only in the ER+ patients but not the ER- patients. **B.** Validation with ER+ and ER- patients in the Curtis breast cancer cohort confirms that *SERPINA1* has a significant predictive value in the ER+ but not the ER- patients.

Based on our findings that the expression of *SERPINA1* is regulated by both ER and HER2, we then performed the Kaplan Meier survival analysis by dividing the patients into high and low expression groups based on the median of the single gene *SERPINA1*. We found that in the TCGA training cohort with 570 ER+ patients, this gene showed a statistically significant predictive value (*p* = 0.0002) (Figure 5A). In contrast, the same analysis performed on the ER- patients was not statistically significant (Figure 5A), confirming that it is an ER-regulated gene. We validated this finding in the Curtis cohort using Disease Free Survival (DFS) analysis with 986 ER+ patients (*p* = 0.01) (Figure 5B) which correlated with OS to serve as strong prognostic factor for patient treatment outcome.

**Figure 6:**
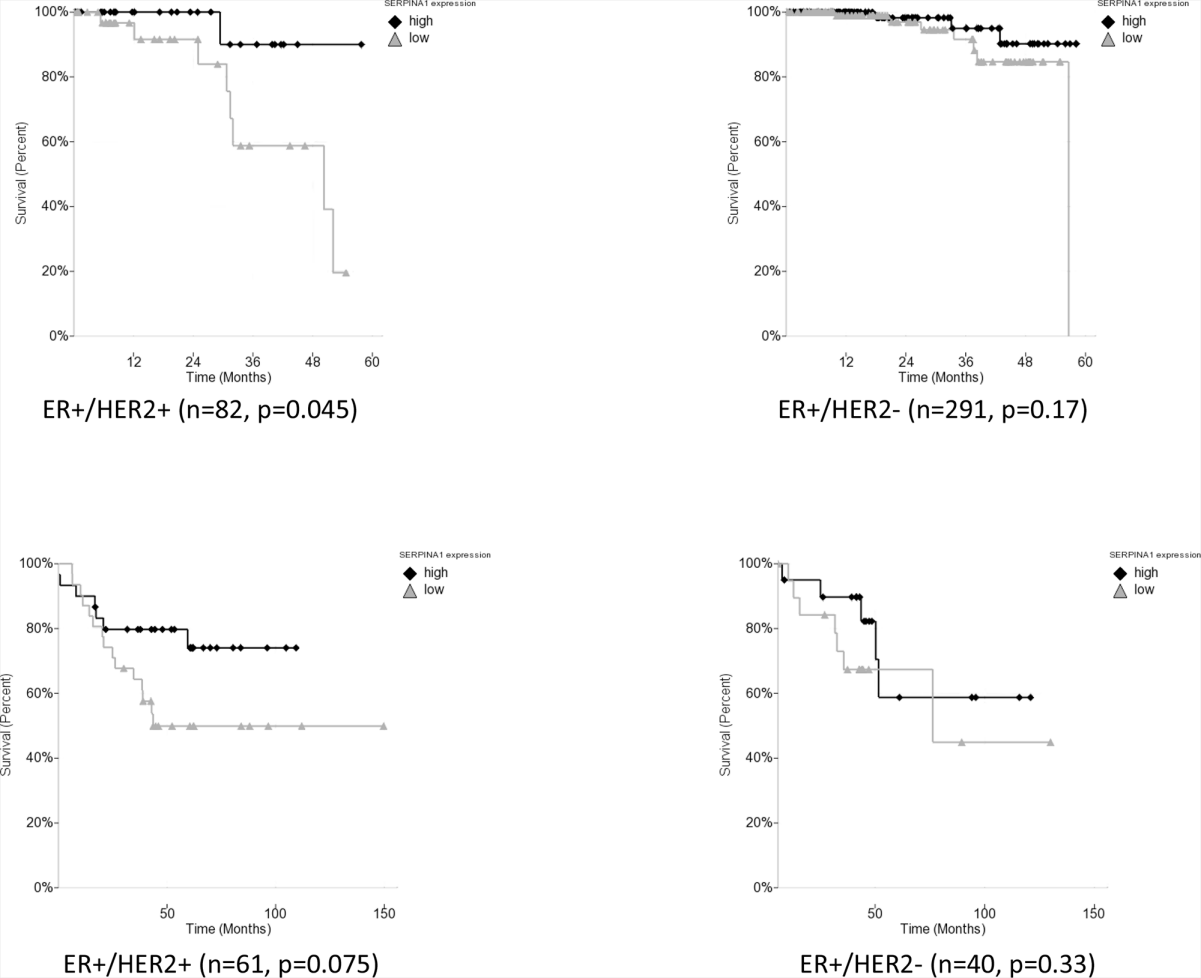
Survival analysis of *SERPINA1* in TCGA and Bild breast cancer patient cohorts with ER+/HER2+ status **A.** *SERPINA1* has a significant predictive value in the ER+/HER2+ but not the ER+/HER2- patients from TCGA cohort with OS outcome. **B.** Validation with ER+/HER2+ and ER+/HER2- patients in the Bild cohort. DFS analysis of ER+/HER2+ patients in Bild patient cohort shows that patients with high expression of *SERPINA1* has a better treatment outcome.

To further validate our findings, we decided to carry out the survival analysis in four additional patient cohorts [24–28] (Table 1). However, we failed to observe any correlation between *SERPINA1* levels and survival in other four cohorts of ER+ patients, namely Chin, Pawitan, Desmedt, Sotiriou (data not shown). Since we confirmed that the expression of *SERPINA1* can also be regulated by HER2, we then checked the HER2 status of patients in the six cohorts and found that only the TCGA, Curtis and Bild cohorts had a significant number of HER2-positive patients in ER+ subcohorts, whereas the other 4 cohorts had mostly HER2-negative patients or patients with unknown HER2 status in their ER+ subcohorts (Table 1). Such observations led us to propose that the HER2 status is related to the predictive value of *SERPINA1* on patient survival outcome. To verify this hypothesis, we performed further survival analysis with subgroups of patients by separating the patients based on ER and HER2 status. In the TCGA cohort, we subdivided the ER+ patients based on HER2 status, and found that the *SERPINA1* has a significant predictive value in the ER+/HER2+ group with 82 patients (*p* = 0.045) but not the ER+/HER2- (Figure 6A), ER-/HER2+, or ER-/HER2- patients (ER- data not shown). For validation we used the Bild breast cancer patient cohort with 61 ER+/HER2+ patients [24]. The *p*-value is 0.075 which was slightly above 0.05, perhaps due to the low number of ER+/HER2+ patients (Table 1), but the trend of separation was observed visually (Figure 6B). We performed the same analysis on the Curtis ER+/HER2+ patients, and also observed a visual separation of the two curves, but the curves intersected each other at the earlier timepoints, and the *p*-value was 0.14, so these results were not statistically significant. To further establish the value and uniqueness of *SERPINA1* as a predictive marker, we investigated the predictive ability of some well known ER target genes *TFF1* (pS2), *PGR* and *GREB1*. We performed the survival analyses by grouping the patients in the TCGA cohort based on ER status only, and both ER and HER2 status (Supplementary Figure 3). In ER+ and ER+/HER2+ patients, the 3 genes were not able to separate the patients into high and low risk groups. This further supports the unique ability of *SERPINA1* to predict patient survival, because *SERPINA1* is regulated by both ER and HER2.

**Table 1:** Summary of six patient cohorts tested for patient survival analyses

Cohort	Total # patients	# of ER+ patients	# of ER+ patients HER2 + / − / Unknown
TCGA	779	570	88 / 318 / 164
Curtis	1284	986	77 / 909 / 0
Bild	170	114	61 / 40 / 13
Chin	117	74	4 / 45 / 25
Desmedt	198	134	No HER2 status data
Pawitan	159	62	0 / 62 / 0
Sotiriou	99	65	No HER2 status data

TCGA, Curtis and Bild cohorts have a significant number of HER2 positive patients. The other 4 cohorts have mostly HER2 negative or HER2 status unknown patients.

## DISCUSSION

ER is a key player in estrogen (or hormone)-dependent breast cancer, and its action can be modified through many mechanisms (see a recent review by Manavathi et al. 2013) [34]. Ross-Innes et al [35] provided an excellent example to show that changes in ER binding is associated with clinical outcome in breast cancer. There have been extensive studies of ER binding in estrogen-responsive cells/tissue through ChIP-on-chip and ER ChIP-seq analyses [30, 36–39]. From our Illumina ChIP-seq and Affymetrix GeneChip microarray data, it is clear that the ER in the LTEDaro cells behaved differently from that in the MCF-7aro cells. The analysis of distance to transcription start site (TSS) showed that in the estrogen-responsive MCF-7aro cells, the ER recruitment proximal to the TSS was dependent on E2, but in the resistant cells the ER recruitment to the same region could occur without E2. Comparison of the intensity and number of peaks revealed that in the MCF-7aro cells most of the ER binding were very weak without E2, and the ER binding was greatly enhanced when E2 was present. On the other hand, in the LTEDaro DMSO cells, a significant number of ER binding sites could be detected. Our ER binding data supports previous proliferation studies generated from this and other laboratories [40]. In the MCF-7aro cells, proliferation is entirely dependent on the E2-mediated activation of ER, and other growth factor pathways are not essential for the proliferation of these cells. LTEDaro cells are still dependent on the ER pathway for proliferation, as indicated by the fact that fulvestrant (ICI 182, 780) was able to partially suppress the proliferation of LTEDaro [5]. However, several signal transduction pathways were found to be activated and crosstalk with ER in this AI-resistant line [5]. HER2 is one of the important signaling proteins that play a role in the phosphorylation of ER, as in luminal B breast cancer [41]. In our laboratory, we generated an MCF-7 cell line that over-expresses aromatase and HER2, i.e., HER2-aro, and showed that this line was resistant to both AIs and ICI [5]. Addition of E2 to LTEDaro provided additional ER binding sites (data not shown). The physiological significance of E2 induced ER binding in LTEDaro E2 requires further molecular characterization.

Through the bioinformatics analysis in combination with gene expression microarray data, we identified *SERPINA1* as one such ER target gene that clearly had E2-dependent ER binding in MCF-7aro cells and stronger E2-independent ER binding in LTEDaro cells. Its expression in both types of cells was significantly suppressed by the treatment of ER degrader, fulvestrant. Here we confirmed that *SERPINA1* is a direct ER target gene, as supported by our ER ChIP-seq, ChIP PCR validation, microarray, gene expression qPCR data, and siRNA results. Considering the fact that *SERPINA1* is highly expressed in LTEDaro, its expression must be up-regulated by ER through cross-talk with growth factor-regulated pathways. From searching the GEO data, we found that *SERPINA1* is also a HER2-regulated gene. This was supported by the fact that HER2-aro cells had much higher expression levels of *SERPINA1* than MCF-7aro cells. The expression of *SERPINA1* in these resistant cells was reduced upon the treatment of HER2 siRNA (Figure 4B). Based on our results, we hypothesize that in LTEDaro and HER2-aro cells, ER is activated through phosphorylation by signaling pathways activated by HER2 [5] and *SERPINA1* is a unique gene whose expression can be induced by phosphorylated ER.

As a translational research project, this is a good example how we can correlate our ER binding data to patient outcome information using bioinformatics analysis. Our attempts pointed out some limitations in such effort. Our Kaplan Meier survival analysis found that *SERPINA1* expression had a significant association with patient survival in ER+ patients using TCGA and Curtis cohorts, and with ER+/HER2+ patient survival in the TCGA and Bild cohorts. However, we were not able to reach the same conclusion using four other patient cohorts (i.e., Chin, Pawitan, Desmedt, Sotiriou). This could be due to differences in the cohort size and composition of patient population of ER/PR/HER2 status in these cohorts. This experience pointed out the need of large cohorts with detailed clincopathological features and treatment outcome information for more meaningful prediction. Since the majority of breast cancer patients are ER+, our finding that *SERPINA1* can predict survival in ER+ patients suggests that it could be a potential prognostic marker, and many patients may benefit from this additional knowledge. Although the results were not expected originally, we are excited about the potential predictive value of *SERPINA1* transcript expression levels in the ER+/HER2+ breast cancer, since these patients have relatively poor prognosis.

As two major regulatory pathways, ER and HER2 cross-talk when they co-exist [42, 43]. Approximately 10 percent of breast cancer patients are ER+ and HER2+, and these patients have worse survival compared to ER+/HER2- patients [44]. The ER+/HER2+ breast cancer is an important subtype of luminal B breast cancer [41]. It has been also observed that a significant number of recurring tumors from luminal A cancer are converted to luminal B HER2+ [41]. Overexpression of HER2 in ER+ breast cancer is well recognized to reduce the effectiveness of endocrine therapy, as observed preclinically [5] and clinically [45]. Similarly, co-expression of ER is known to result in a poor trastuzumab response [46]. Previous studies have shown that in HER2+ breast tumors, the mRNA levels of HER2 is correlated with pathological complete response (pCR) rate only in ER+ patients but not in ER- patients [47]. Extensive studies have been performed to demonstrate how ER-regulated pathways and HER2-regulator pathways can modulate each other [43]. Preclinical experiments from our laboratory have found that LTEDaro, an AI-resistant model, is still partially responsive to ICI, but HER2-aro, a HER2-overexpressing line, fails to respond to either AI or ICI. These studies point out that in ER+/HER2+ cancer, it is essential to suppress both regulatory pathways, possibly additional mechanisms regulated both of these pathways.

While the expression level of *SERPINA1* was found to be higher in endocrine resistant cells than responsive cells, unexpectedly, the Kaplan Meier survival analysis revealed that high expression of this gene associated with better survival in ER and HER2 positive luminal B subtype of breast cancer. To explain our findings, we hypothesize that a high expression of *SERPINA1* indicates the important roles of ER and HER2 in driving the growth of the tumors. Therefore, in ER+ breast cancers, the expression of *SERPINA1* could be an indication of estrogen-mediated ER activation and its expression levels correlate to the survival. A high expression of this gene is thought to be a strong indicator for the cooperative activation by both signaling pathways and to be a “good” response to both anti-ER and anti-HER2 therapies. Many ER-regulated genes, such as *TFF1*, *PGR* and *GREB1*, are known to be induced in endocrine-resistant cancer, but did not show statistical significant correlation with survival outcome based on our analysis.

In early stage breast cancer, women with ER+/HER2+ cancers are treated with adjuvant trastuzumab. Recent data suggests that a subset of these patients may not benefit from trastuzumab [48]. Clinical data has shown that ER+/HER2+ patients generally have worse outcome than ER-/HER2+ or ER+/HER2- patients [17, 18], and a predictive marker to predict a subgroup of patients with better outcome will be valuable. To investigate whether there are any differences in the treatments received by the patients with better survival compared to those with worse survival, we have examined the treatment information of ER+ and HER2+ patients in the TCGA cohort, but such information is difficult to obtain with treatment outcome. We made an attempt to compare the limited treatment information that we have of the patients with high and low levels of *SERPINA1* expression, which corresponds to better and worse survival groups respectively (Supplementary Table 2). There were no major differences in the treatment strategies between good and bad responders, suggesting that *SERPINA1* could be an outcome predictor independent of treatment options. Chemotherapy and HER2-directed therapy was widely used as systemic treatment for patients with ER+ and HER2+ disease. Anti-HER2 therapy has been shown to improve endocrine therapy in ER+/HER2+ positive cancer, as demonstrated in preclinical models [49, 50]. A recently completed trial revealed that a combination of anti-HER2 therapy and endocrine therapy could be valuable to treat ER+/HER2+ patients [51]. Furthermore, from the EGF30008 and TAnDEM (TrAstuzumab in Dual HER2 ER-positive Metastatic breast cancer) trials, lapatinib + letrozole and trastuzumb + anastrozole were shown to improve time to progression versus AI monotherapy, respectively [52]. A detailed analysis was reported by Delea et al. [52] that lapatinib + letrozole was not likely to be cost-effective than trastuzumab + anastrozole. Therefore, for those ER+/HER2+ patients with high levels of *SERPINA1* expression, a less toxic or more cost-effective treatment may be also considered.

In conclusion, this is a translational research study. Attempts are made to translate results from ER ChIP-seq analysis to breast cancer patient outcome information. Ross-Innes et al. [35] have provided a strong precedence that differential ER-binding is associated with clinical outcome in breast cancer. Based on our findings, we propose that the expression of *SERPINA1*, an ER and HER2 regulated gene, is linked to the outcome of ER+ and ER+/HER2+ breast cancer.

## MATERIALS AND METHODS

### Cell lines

The MCF-7aro cell line was generated as a model to study the action of AIs [3]. The LTEDaro cell line was generated by a long-term estrogen deprivation of MCF-7aro and is used as a model of the late stage of endocrine resistance [4]. HER2-aro is a MCF-7 line that over-expresses HER2 and aromatase [5] and is a model of *de novo* AI resistance as well as a model of luminal B, HER2-overexpressing subtype.

### ER ChIP-seq analysis

MCF-7aro and LTEDaro cells were cultured in hormone-free MEM for 5 days and serum-free MEM for 1 day. When the cell number reached about 1 × 10^7^, both the LTEDaro and MCF-7aro cell lines were serum starved for 24 hours followed by treatment with 100 nM E2 or DMSO vehicle for 45 minutes, and cross-linked with 1% formaldehyde at room temperature for 10 minutes. Cells were enlarged in hypotonic buffer and nuclei were isolated by addition of NP-40 and centrifugation. The chromatin was sonicated to yield a majority of fragments with sizes between 100–300 base-pairs (bp). ERα antibodies (HC-20; sc-543) and IgG antibodies (sc-2027) from Santa Cruz Biotechnologies (Santa Cruz, CA) were used for the immunoprecipitation and control respectively. The enriched chromatin was purified with the Qiagen Minelute PCR purification kit (Valencia, CA) and prepared for high-throughput sequencing.

The purified ChIP DNA samples were sequenced, using Illumina Solexa Genome Analyzer II (San Diego, CA) at the DNA sequencing core facility (City of Hope, Duarte, CA), to generate short reads that are 36 to 45 bp in length. The short reads were mapped to human genome (Hg18) using the Bowtie [20] alignment tool. Peak-calling software, MACS v1.4.1 [21], was used to detect binding sites using the alignment results by setting a statistically significant cutoff (*p*-value = 1.00e-5) comparing the ER versus IgG sample.

### *SERPINA1* promoter cloning and luciferase activity assays

The SERPINA1 promoter region was cloned into the pGL3 luciferase vector from Promega (Madison, WI). From the ChIP-seq peak calling data, the ER binding site covers a region of approximately 2 kb which overlaps with the TSS. The predicted ERE motif lies near the center of this binding region and is 19 bp in length. We cloned a region that covers the TSS and upstream 2 kb region, with a total length of 2.1 kb, which contains the full length *SERPINA1* promoter. We also generated truncated versions of this promoter, with and without the predicted ERE motif, which are 270 bp and 240 bp in length respectively (Figure 3A). MCF-7aro and LTEDaro cells were then transfected using X-treme gene HP reagent (Roche, Indianapolis, IN) and assayed for luciferase activity.

### Overlap analysis of ER binding sites

The ER binding sites from MCF-7aro E2 and LTEDaro DMSO were selected by FDR ≤ 0.5%, and the sites were labeled as “common” if there was at least 1 bp overlap, and the remainder sites were labeled as “unique”. The ER binding sites associated with resistant cells were identified by comparing normalized binding site intensities of LTEDaro DMSO over MCF-7aro E2 with a change ratio ≥ 0.9 as the cutoff. A positive fold change of ≥ 0.9 represents LTEDaro DMSO binding sites that have 90% or greater binding intensity compared to MCF-7aro E2. This group of ER binding sites that are important in resistant cells was annotated with genes within +/−20 kb, and were then integrated with the gene expression data from our previous microarray study [1]. The genes were filtered based on a cutoff of 1.2 fold change with FDR adjusted *p* < 0.05, and there were 350 genes that passed all filters.

### Kaplan-Meier survival analysis

To identify genes with potential survival predictive power, the 350 genes from the ER-binding site overlap analysis were ranked based on Cox scores, which represents the association of gene expression in patient cohorts with patient survival data. For a single gene survival correlation, patients were grouped as high expression and low expression subgroups based on the median expression of that gene. For a group of genes, patients were grouped as High-Risk and Low-Risk subgroups based on 2-means clustering of the selected significant genes for Kaplan-Meier survival analysis [22]. Cox scores were calculated using R Bioconductor v3.0, and 2-means clustering analysis was performed in Partek Genomics Suite 6.6. Kaplan-Meier survival analysis was then used to determine the survival differences between the High-Risk and Low-Risk subgroups with *p*-values calculated by log-rank test in Partek Genomics Suite 6.6. Based on the study by Bair and Tibshirani, we used a Cox score cutoff of 2.39 to select top genes with better survival correlation [22], resulting in a list of 35 genes. Using TCGA breast cancer patient cohort as the training set [6], we performed further analysis and discovered a single gene, and validation was performed in the Curtis and Bild cohorts [23, 24]. In addition to the TCGA, Curtis and Bild cohorts, we have performed the survival analysis on four other patient cohorts, namely Chin, Desmedt, Pawitan, and Sotiriou [25–28].

### Semi-quantitative PCR analysis of ER binding

ChIP DNA was prepared as described above. Primers were designed to amplify a 166bp region overlapping with the center of the peak as detected by ChIP-seq. The PCR was performed for 30 cycles using Promega GoTaq Green mastermix (Madison, WA) and analyzed by agarose gel electrophoresis.

### Quantitative PCR analysis of *SERPINA1* expression

For gene expression quantification, MCF-7aro LTEDaro, HER2-aro, BT-474 and MDA-MB-361 cells were treated for 24 hours with 1 nM E2, 200 nM ICI 182780, and/or HER2 siRNA. RNA was extracted from cells with Trizol reagent, and cDNA was synthesized with SuperScript III system from Invitrogen (Grand Island, NY). Quantification of cDNA was performed using the Bio-rad iQ5 system. For the gene expression analysis, the delta Ct method was used, with β-actin as the normalizer, and the gene expression values were calculated relative to the DMSO control. Primer sequences are provided in the Supplementary Table 1. *SERPINA1* gene expression primer sequences have been previously published [8].

## SUPPLEMENTARY FIGURES AND TABLES


