# Supplementary material for: SERPINA1 is a direct estrogen receptor target gene and a predictor of survival in breast cancer patients
Source: Oncotarget. 2015 Jun 29;6(28):25815–27. doi: 10.18632/oncotarget.4441 (PMC4694868; doi:10.18632/oncotarget.4441)
Supplement: Supplementary file 1 [file oncotarget-06-25815-s001.pdf]

SUPPLEMENTARY FIGURES AND TABLES

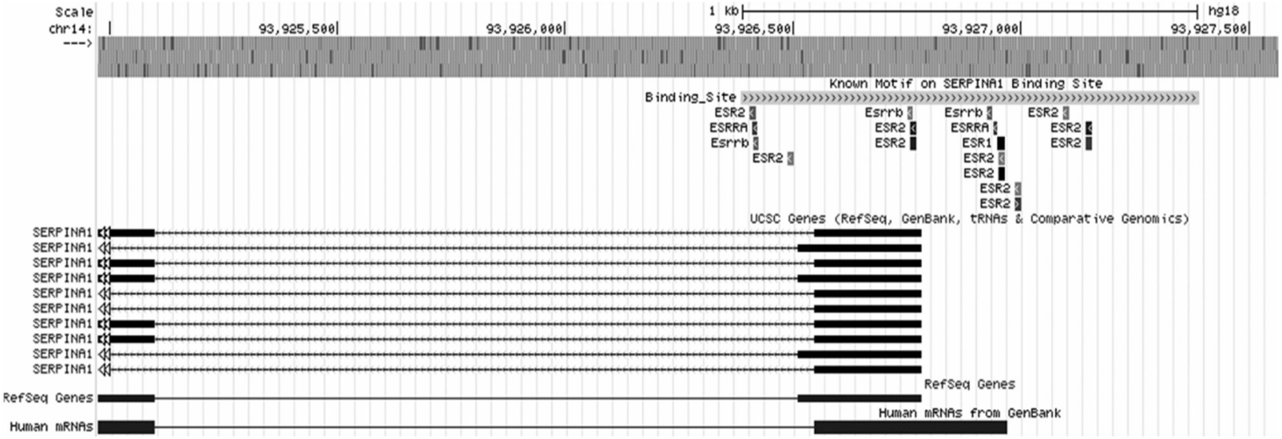

Supplementary Figure 1: ER binding site at the *SERPINA1* promoter contains an ERE. Known motif mapping of the ER binding site in the *SERPINA1* promoter reveals the ERE motif in the center of the binding sites.

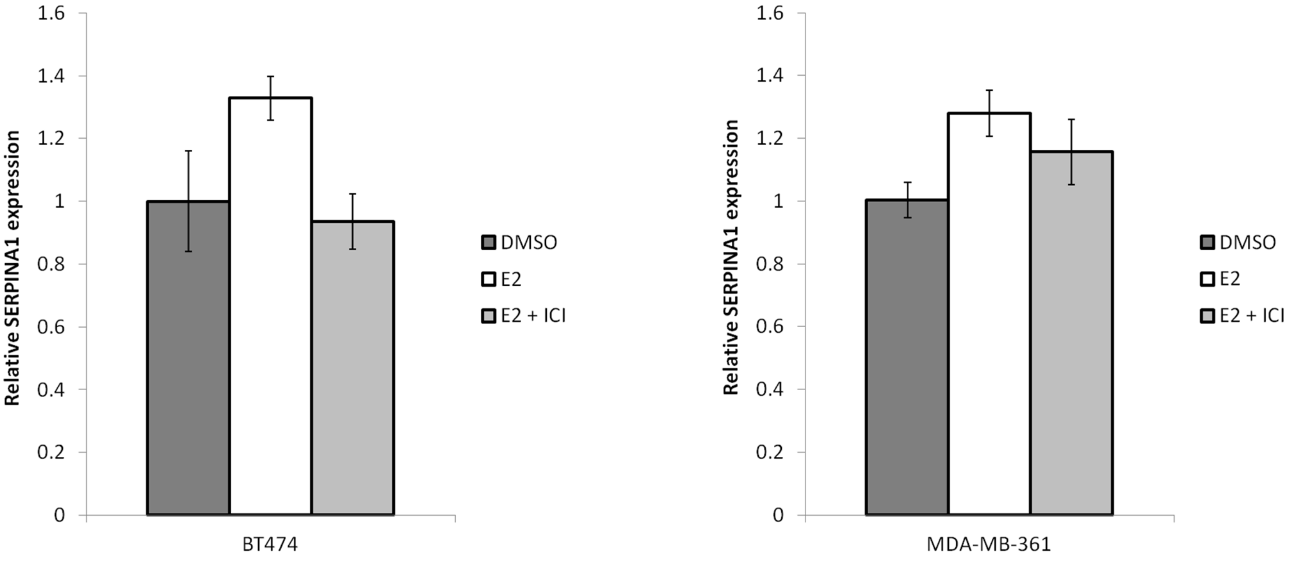

Supplementary Figure 2: SERPINA1 expression in BT-474 and MDA-MB-361 cells. Quantitative PCR analysis of gene expression with E2 or ICI treatment shows no significant change in *SERPINA1* levels.

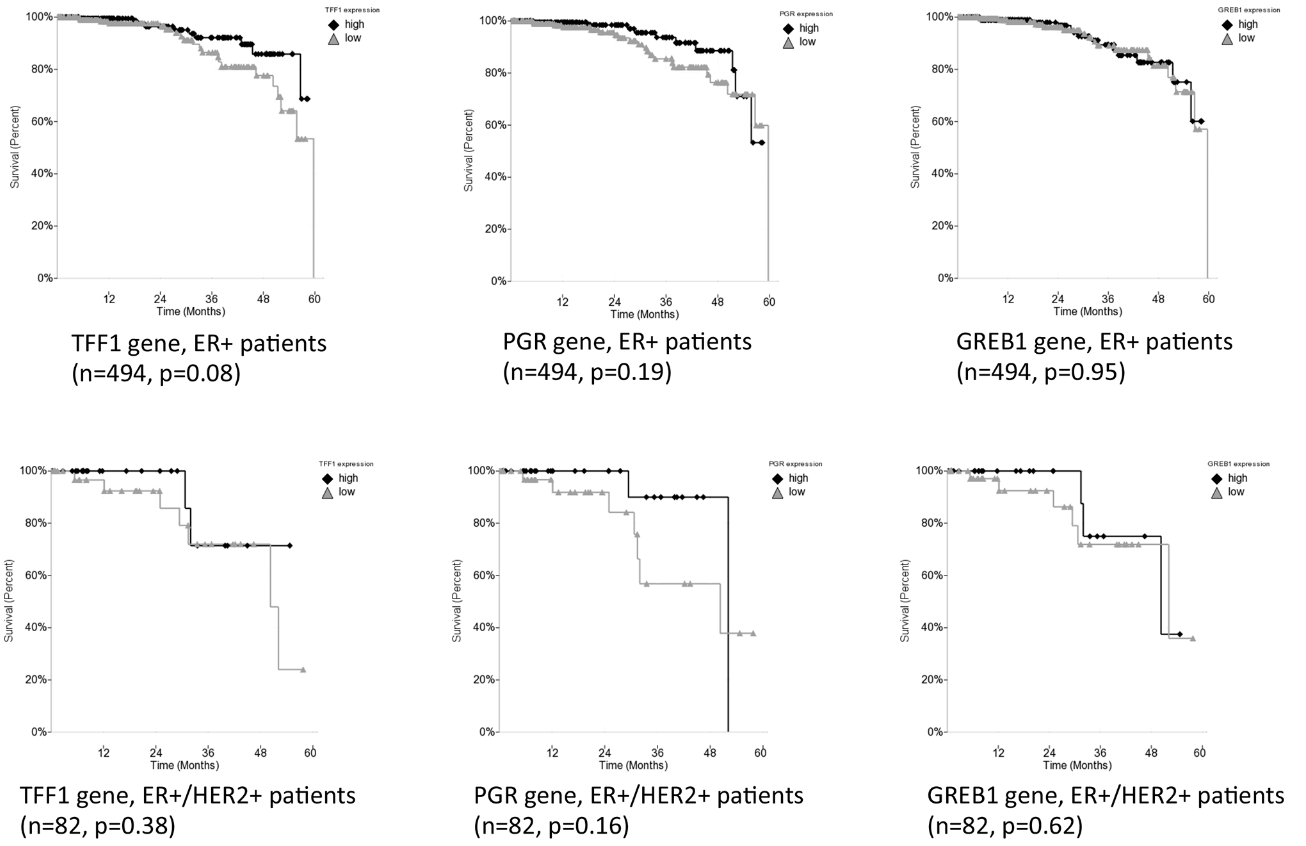

Supplementary Figure 3: Overall Survival in TCGA ER+ and ER+/HER2+ patients with high and low expression of 3 common ER target genes: TFF1, PGR, GREB1. Kaplan Meier survival analyses of the 3 genes TFF1, PGR, and GREB1 in ER+ and ER+/HER2+ patients shows that none of these genes have a significant prediction of overall survival.

Supplementary Table 1: Primer sequences for ChIP and gene expression analyses. Sequences of the primers used for the validation of ER binding and gene expression of *SERPINA1* and *TFF1*.

Target Name	Primer Sequence (5′ – 3′)
SERPINA1 F ChIP	GCCCGGCATGTCACCTGTTGTA
SERPINA1 R ChIP	CCTGCCAGTTATTGGTGCCAGGT
SERPINA1 F expression	CACCGTGAAGGTGCCTATGATG
SERPINA1 R expression	GGCATTGCCCAGGTATTTCATC
TFF1 F ChIP	TTCATGAGCTCCTTCCCTTC
TFF1 R ChIP	ATGGGAGTCTCCTCCAACCT
TFF1 F expression	AACAAGGTGATCTGCGCCCTG
TFF1 R expression	GGCGTGACACCAGGAAAACCA

Supplementary Table 2: Summary of drug treatment information in TCGA patients. Using the available drug information in TCGA patient cohort, we compared the treatments received by the patients with high and low *SERPINA1* expression, and found no significant difference.

Drug class	Drug name	Number of patients	SERPINA1 high	SERPINA1 low
Chemotherapy	5-Fluorouracil	4	3	1
Chemotherapy	ac	1	1	0
Chemotherapy	Adriamycin	5	1	3
Chemotherapy	adriamycin+cyclophosphamide	1	0	1
Endocrine therapy	Arimidex	8	4	4
Endocrine therapy	Aromasin	2	2	0
Chemotherapy	Carboplatin	3	0	3
Chemotherapy	Cyclophosphamide	6	1	2
Chemotherapy	Cyclophosphane	4	3	1
Chemotherapy	Cytoxan	7	2	4
Chemotherapy	Docetaxel	3	1	2
Chemotherapy	Doxorubicin	9	4	2
Chemotherapy	doxorubicin+cyclophosphamid	1	0	1
Endocrine therapy	Exemestane	1	1	0
	Herceptin	9	4	5
	Lapatinib	2	0	2
Endocrine therapy	Letrozole	2	1	1
Endocrine therapy	Lupron	1	0	0
Chemotherapy	Paclitaxel	5	1	1
Chemotherapy	Paclitaxel (Protein-Bound)	1	1	0
Endocrine therapy	Tamoxifen	14	6	4
Chemotherapy	Taxol	4	2	2
Chemotherapy	Taxotere	5	2	3
	Trastuzumab	4	2	1
Endocrine therapy	Zoladex	2	1	1
	Zometa	1	1	0
	**Total number of treatments:**	**105**	**44**	**44**
	**Endocrine therapy:**		**15**	**10**
	**Chemotherapy:**		**22**	**26**
